# Probing strong first-order electroweak phase transition scenarios in two-Higgs-doublet model with FCC-ee/CEPC

**DOI:** 10.1140/epjp/s13360-026-07792-5

**Published:** 2026-05-26

**Authors:** Anisha, Francisco Arco, Stefano Di Noi, Christoph Englert, Margarete Mühlleitner

**Affiliations:** 1 Institute for Theoretical Physics, Karlsruhe Institute of Technology, 76131 Karlsruhe, Germany; 2 Institute for Astroparticle Physics, Karlsruhe Institute of Technology, 76344 Eggenstein-Leopoldshafen, Germany; 3 Deutsches Elektronen-Synchrotron DESY, 22607 Hamburg, Germany; 4 Department of Physics and Astronomy, University of Manchester, Manchester, M13 9PL UK

## Abstract

We investigate the potential of future electron–positron colliders, such as FCC-ee and CEPC, to test a strong first-order electroweak phase transition (SFOEWPT) in the CP-conserving type I two-Higgs-doublet model. We confront the SFOEWPT-favored parameter space with projected electroweak precision and Higgs measurements, as well as searches for additional scalar states. We show that radiative corrections to $e^+e^-\rightarrow hZ$ production can generate deviations in the cross section that exceed the anticipated sub-percent precision at lepton colliders, even when LHC and Z-pole measurements remain consistent with the SM. Precision Higgs-strahlung measurements therefore provide an indirect and sensitive probe of electroweak-scale BSM effects.\query{Please check the edit made in the article title.}

## Introduction

Despite the remarkable success, the Standard Model (SM) cannot account for the observed baryon–antibaryon asymmetry of the Universe (BAU). Although it satisfies the Sakharov conditions [2], it fails to generate a strong first-order electroweak phase transition (SFOEWPT) for the observed Higgs mass [3, 4], as required for electroweak baryogenesis (EWBG). This strongly motivates physics beyond the SM. A minimal and well-studied extension is the two-Higgs-doublet model (2HDM) [5–7], in which additional scalars can induce the necessary potential barrier through loop effects.

With no direct evidence for new physics at the Large Hadron Collider (LHC), indirect probes through precision measurements have become increasingly important. While the High Luminosity LHC (HL-LHC) will further improve sensitivity, future electron–positron colliders such as FCC-ee [8] and CEPC [9] will provide unprecedented precision in electroweak measurements. In this proceedings contribution, based on Ref. [1], we investigate the type I 2HDM parameter space compatible with an SFOEWPT and examine the impact of precision *Z*-pole and $e^+ e^- \rightarrow hZ$ measurements at FCC-ee.

## The 2HDM and electroweak phase transition

**Model description**: We consider the CP-conserving 2HDM [5–7] with a discrete $\mathbb {Z}_2$ symmetry. The physical scalar spectrum contains five Higgs bosons: two neutral CP-even states *h* and *H*, one CP-odd state *A*, and two charged states $H^\pm$. The mixing angles $\alpha$ and $\beta$ diagonalize the CP-even sector and CP-odd/charged sectors, respectively. We focus on the type I, where all fermions couple to the same Higgs doublet. The input parameters are

1 $$\begin{aligned} v\,, \; m_h\,, \; m_H\,, \; m_A\,, \; m_{H^\pm }\,, \; \tan \beta \,, \; \cos \!\left( \beta -\alpha \right) \,, \; \bar{m} = m_{12}/\sqrt{\sin \beta \cos \beta }\,, \end{aligned}$$

with $v\simeq 246\ \textrm{GeV}$, the SM vacuum expectation value (vev). Assuming that *h* is the observed SM-like Higgs ($m_h<m_H$), agreement with LHC signal strength constraints can be achieved either in the *alignment limit*, $\cos \!\left( \beta -\alpha \right) \rightarrow 0$, where the tree-level couplings of *h* approach their SM predictions, or in the *decoupling limit*, in which $\cos \!\left( \beta -\alpha \right) \rightarrow 0$ and the additional scalars are much heavier than the electroweak scale, $m_H,\, m_A,\,m_{H^\pm }\gg v$.

**SFOEWPT in 2HDM**: To study the electroweak phase transition, we consider the one-loop finite-temperature effective potential

2 $$\begin{aligned} V_{\textrm{eff}}\!\left( T\right) = V_{\textrm{tree}} + V_{\textrm{CW}} + V_{\textrm{CT}}+V_{\textrm{T}}\!\left( T\right) + V_{\textrm{daisy}}\!\left( T\right) \,, \end{aligned}$$

where, $V_{\textrm{tree}}$ is the tree-level potential, $V_{\textrm{CW}}$ the Coleman–Weinberg contribution [10], $V_{\textrm{CT}}$ the finite counter-term, $V_{\textrm{T}}$ the thermal corrections evaluated in the high-temperature limit [11, 12] and $V_{\textrm{daisy}}$ the resummed daisy corrections following Arnold–Espinosa prescription [13]. The numerical analysis is performed using $\texttt{BSMPTv3}$ [14–16]. The strength of the transition is evaluated at the percolation temperature $T_{p}$ with the corresponding Higgs vev $v_{p}$, requiring $\xi _p\equiv v_p\!\left( T_p\right) \big /T_p>1$ for a strong electroweak phase transition. The electroweak phase transition in the 2HDM has been extensively studied (see, e.g., in Refs. [17–24]). An SFOEWPT requires relatively light BSM scalars and sizeable radiative corrections that generate a barrier between the symmetric and broken phases and is therefore incompatible with the decoupling limit.

**Parameter scans**:  We perform a random scan over the input parameter ranges

3 $$\begin{aligned} \begin{gathered} m_H \in \left[ 150,\, 1500\right] \ \textrm{GeV}\,, \quad m_A,\ m_{H^\pm } \in \left[ 20,\, 1500\right] \ \textrm{GeV}\,, \quad \\ \tan \beta \in \left[ 0.5,\, 50\right] \,, \quad \cos \!\left( \beta -\alpha \right) \in \left[ -0.35,\, 0.35\right] \,, \quad \bar{m} \in \left[ 0,\, 1500\right] \,, \end{gathered} \end{aligned}$$

fixing $m_h=125.25$ GeV. The sampled points are tested with ScannerS [25, 26] for theoretical consistency (perturbative unitarity, boundedness from below and a global electroweak minimum) and agreement with current experimental constraints. The latter include electroweak precision constraints (requiring $S,\, T,\,U$ to lie within the $2\sigma$ region of SM EW fit [27]), LHC Higgs measurements and the searches for additional scalars via HiggsTools [28], as well as the flavor constraints at the 95% CL [27]. For the surviving points, we compute the thermal history using BSMPTv3, ensuring next-to-leading order potential stability and that the broken electroweak vev is realized at $T=0$.

## Testability of the SFOEWPT region at colliders

In this section, we test the SFOEWPT allowed parameter region against the projected sensitivities at FCC-ee. We also compare with the HL-LHC $\kappa$-modifier projections and comment on the sensitivity of LHC searches for additional BSM scalars.

**Fig. 1:**
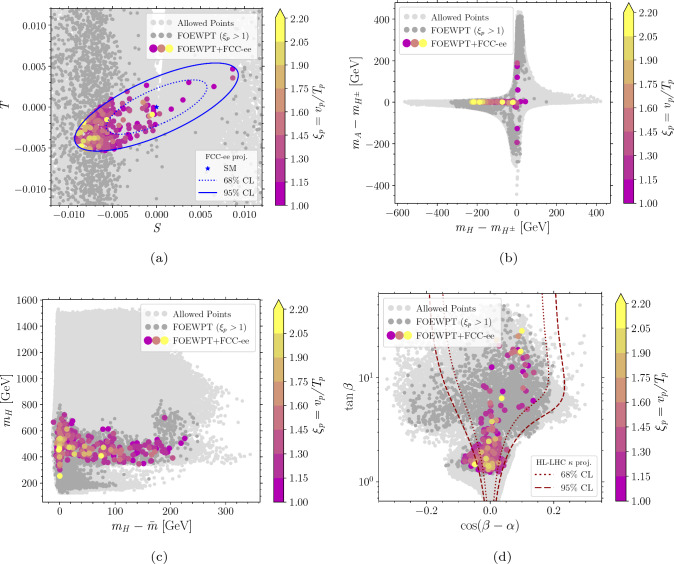
Projected FCC-ee *Z*-pole sensitivity to the SFOEWPT scan. Panel 1a: *S* and *T* ellipses (68% CL dotted, 95% CL solid). Panel 1b and 1c: allowed mass spectra. Panel 1d: HL-LHC Higgs signal strength projections (68% CL dotted red, 95% CL dashed red) in the $\tan \beta$ versus $\cos (\beta -\alpha )$ plane

**Fig. 2:**
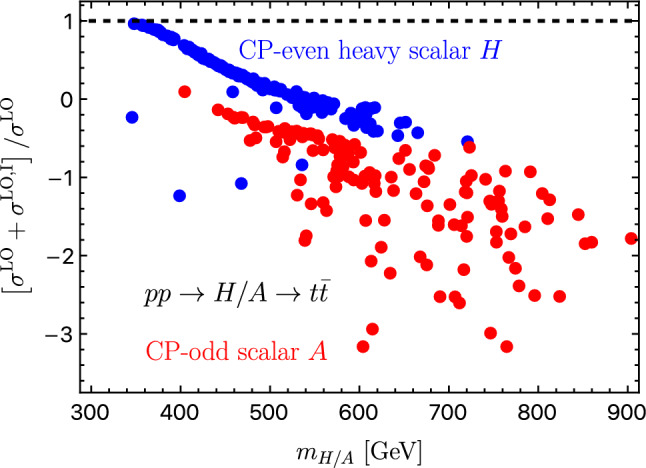
Interference-corrected LHC cross sections for *H* (blue) and *A* (red), as a function of the mass, compared to the pure signal. Points correspond to SFOEWPT preferred scenarios

**Fig. 3:**
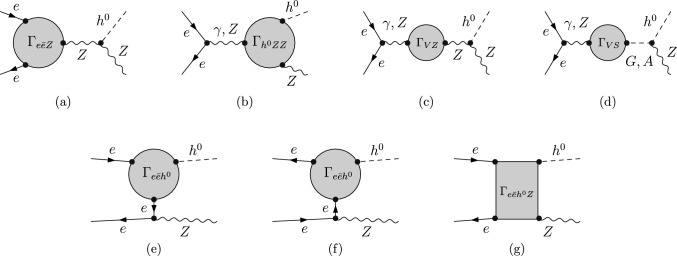
Different topologies contributing at NLO to $e^+ e^- \rightarrow hZ$ in 2HDM

**Fig. 4:**
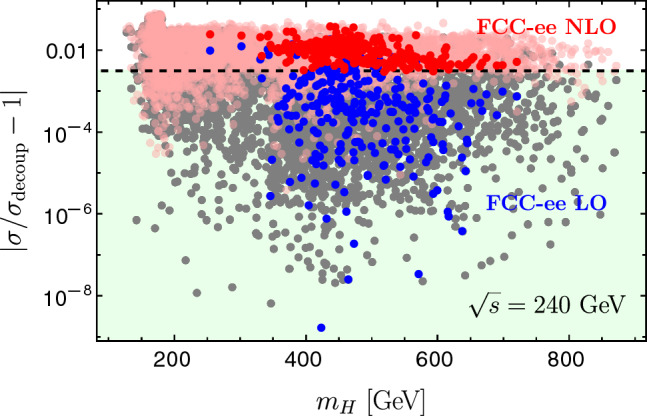
FCC-ee *hZ* production cross section at $\sqrt{s}=240~\text {GeV}$ relative to the decoupling limit, including projected *Z*-pole sensitivity. Blue (red) points denote LO (NLO) predictions and the green region is the projected FCC-ee sensitivity $\Delta \sigma /\sigma =0.31\%$. Dark gray (pink) points denote LO (NLO) sensitivity without imposing FCC-ee *Z*-pole constraints. All points satisfy the SFOEWPT requirement

- **FCC-ee** *Z***-pole constraints**: The first stage of FCC-ee (or CEPC) is a *Z*-pole run at $\sqrt{s}=m_{Z}$. We compare the SFOEWPT preferred 2HDM parameter space with the projected electroweak precision constraints at FCC-ee. The projected $1\sigma$ sensitivities (neglecting SM theory uncertainties) to the Peskin–Takeuchi *S* and *T* parameters [29, 30], including the correlation coefficient $\rho _{ST}$, are
  4 $$\begin{aligned} \sigma _S=0.0038\,, \quad \sigma _T=0.0022\,, \quad \rho _{ST}=0.724\,. \end{aligned}$$
  The allowed regions are shown in Fig. 1a at 68% CL (dotted blue) and 95% CL (solid blue). Here, light gray points satisfy the ScannerS constraints detailed in Sect. 2, dark gray points additionally fulfill the SFOEWPT condition $\xi _p>1$, and colored points survive the projected FCC-ee electroweak precision constraints at 95% CL. The region compatible with both the *Z*-pole projections and an SFOEWPT is smaller than that favored by SFOEWPT alone. Moreover, the surviving points shift toward smaller mass splittings among the BSM scalars, as illustrated in Fig. 1b. This is in direct tension with the sizeable mass splittings between $m_{H}$ and $m_{A}/m_{H^{\pm }}$ required to realize an SFOEWPT in the 2HDM.
- **Comparing FCC-ee and HL-LHC constraints**:  To compare the above sensitivities with those of the HL-LHC, we consider the projected $\kappa$-modifiers for bottom and top quarks, *W* and *Z* bosons, gluons, $\tau$ and $\mu$ leptons from Ref. [31]. The resulting regions are shown in Fig. 1d at $68\%$ (dotted red) and $95\%$ (dashed red) CL limits. The *Z*-pole FCC-ee constraints are more stringent than the HL-LHC projections, particularly for regions far from alignment. In other words, FCC-ee precision measurements push the SFOEWPT-favored region closer to alignment i.e., toward $\cos (\beta -\alpha ) \rightarrow 0$.
- **LHC exotic scalar searches**:  Figure 1b -1c shows that the explored BSM scalar masses lie within the kinematic reach of LHC resonant searches particularly for $m_{A/H}$ above the $t\bar{t}$ threshold. ATLAS and CMS search for heavy CP-even and CP-odd scalars in the $t\bar{t}$ channel [32–34]. However, these searches are strongly affected by signal–signal and signal–background interference effects [35, 36], which can substantially reduce the sensitivity.^1^ Focusing on $gg \rightarrow H/A \rightarrow t\bar{t}$, the leading order signal and interference cross sections are given by
  5 $$\begin{aligned} \hbox {d} \sigma ^{\text {LO}} \sim |{\mathcal {M}}_S|^2, \quad \quad \quad \hbox {d} \sigma ^{\text {LO,I}} \sim 2\, \text {Re} \left( {\mathcal {M}}_S^*{\mathcal {M}}_{\text {rest}}\right) , \end{aligned}$$
  where ${\mathcal {M}}_{\text {rest}}$ includes the QCD continuum $t\bar{t}$ background and contribution from the other heavy scalar. For the parameter points discussed in Sect. 2, the interference corrected cross sections in Fig. 2 show predominantly destructive effects. This indicates that the corresponding parameter region may not be fully excluded at the LHC, despite being kinematically accessible. In such a pessimistic scenario where no significant sensitivity is achieved at the LHC or HL-LHC, we next investigate the additional reach of precision *Zh* measurement at FCC-ee.
- **Sensitivity from Higgs-strahlung measurement at the FCC-ee**: To study the impact of FCC-ee precision on *hZ* production, we compute the radiative corrections to $e^{-} e^{+} \rightarrow hZ$ (see Fig. 3) using FeynArts/FormCalc/LoopTools. The next-to-leading order corrections are separated into QED and weak parts to consistently handle ultraviolet (UV) and infrared (IR) divergences. In the QED contribution, a finite electron mass ($m_e \ne 0$) regulates soft and collinear photon emission below a cut-off $\Delta E=30~\text {GeV}$. The virtual photon contributions (see Fig. 3(a)) are regularized by introducing a photon mass which cancels analytically against the real-emission contributions according to Refs. [37–39]. The resulting finite correction depends on $\Delta E$ and is proportional to the Born amplitude. The weak corrections are computed with $m_e=0$ neglecting electron-Yukawa-suppressed terms. We adopt alternative tadpole scheme[40–44] and renormalize the mixing angles using the pinch technique [45] with counter-terms evaluated at on-shell scalar masses. For further details, we refer to Ref. [1]. The impact of the radiative corrections is shown in Fig. 4 with the FCC-ee projected precision at $\sqrt{s}=240~\text {GeV}$ [46]
  6 $$\begin{aligned} \frac{\Delta \sigma (e^+ e^- \rightarrow hZ)}{\sigma (e^+ e^- \rightarrow hZ) } = 0.31\%\,. \end{aligned}$$
  In the decoupling limit, setting $m_H=m_A=m_{H^\pm }=\bar{m}=1.8\, \textrm{TeV}$ and $\cos \!\left( \beta -\alpha \right) =0$, we obtain $\sigma _{\textrm{decoup}}=0.222$ pb at LO and 0.211 pb at NLO. The red (blue) points denote the NLO (LO) predictions satisfying both the SFOEWPT requirement and the FCC-ee *Z*-pole constraints (see colored points in Fig. 1a ). A clear separation between LO and NLO predictions is observed, with NLO deviations exceeding the projected FCC-ee sensitivity, thereby offering discovery potential. The per-mille *Z*-pole constraints are crucial for this separation as relaxing them (dark gray points in Fig. 1a ) makes some NLO deviations (pink) consistent with the LO within the projected FCC-ee precision. This highlights the importance of *Z*-pole run at future electron–positron colliders. These observed NLO deviations arise from the sizeable radiative corrections required to realize an SFOEWPT.

## Summary and conclusions

While substantial efforts are ongoing at LHC to improve sensitivity, we consider a pessimistic scenario in which no significant gains are achieved at LHC or even HL-LHC. In such a case, future precision lepton colliders could offer a complementary probe. In the absence of a direct discovery, new physics is expected to appear indirectly through small deviations from SM predictions. Assuming the realization of an SFOEWPT in type I 2HDM, we have explored the indirect discovery potential of FCC-ee using the projected precision measurements of *Z*-pole and associated Higgs production. We find that the radiative corrections to $e^{-} e^{+} \rightarrow hZ$, consistent with both an SFOEWPT and FCC-ee *Z*-pole constraints, can generate deviations that exceed the FCC-ee sub-percent precision. This establishes Higgs-strahlung at future lepton colliders as a powerful and sensitive probe of electroweak-scale new physics, capable of testing electroweak baryogenesis motivated scenarios beyond the reach of current direct searches.

## Data Availability

The data associated with this work are available upon reasonable request.

## Footnotes

1 Other channels e.g., $t\bar{t}Z$, $t\bar{t}t\bar{t}$ or charged scalar production do not suffer from such interference effects; however, these searches typically yield smaller production cross sections.
